# Chemically Defined *Lactobacillus plantarum* Cell-Free Metabolites Demonstrate Cytoprotection in HepG2 Cells through Nrf2-Dependent Mechanism

**DOI:** 10.3390/antiox12040930

**Published:** 2023-04-14

**Authors:** Raja Rezgui, Ruhi Walia, Jyoti Sharma, Dwinder Sidhu, Khalid Alshagadali, Saumya Ray Chaudhuri, Amir Saeed, Priyankar Dey

**Affiliations:** 1 Department of Medical Laboratory Sciences, College of Applied Medical Sciences, University of Hail, Hail 55473, Saudi Arabia; 2 Medical and Diagnostic Research Centre, University of Hail, Hail 55473, Saudi Arabia; 3 Department of Biotechnology, Thapar Institute of Engineering and Technology, Patiala 147004, Punjab, India; 4 Council of Scientific and Industrial Research (CSIR), Institute of Microbial Technology, Chandigarh 160036, India; 5 Department of Medical Microbiology, Faculty of Medical Laboratory Sciences, University of Medical Sciences & Technology, Khartoum 12810, Sudan

**Keywords:** probiotic, *Lactobacillus*, antioxidant, Nrf2, metabolites, inflammation, oxidative stress, liver

## Abstract

Centering around the concept that metabolites from the gut commensals can exert metabolic health benefits along the gut–liver axis, we tested whether the cell-free global metabolome of probiotic bacteria can exert hepatoprotective benefits against H_2_O_2_-induced oxidative stress. Cell-free global metabolites of *Lactobacillus plantarum* (LPM) were isolated and untargeted metabolomics was performed. The free radical scavenging potentials of LPM were measured. The cytoprotective effects of LPM were tested on HepG2 cells. A total of 66 diverse metabolites were identified in LPM, among which saturated fatty acids, amino acids and dicarboxylic acids were highly enriched. LPM attenuated cell damage, lipid peroxidation and the levels of intracellular cytoprotective enzymes in H_2_O_2_-treated cells. LPM also attenuated H_2_O_2_-induced increased expressions of TNF-α and IL-6. However, the cytoprotective effects of LPM were diminished in cells that were pretreated with a pharmacological inhibitor of Nrf2. Our data collectively indicate that LPM can significantly attenuate oxidative damage to HepG2 cells. However, the cytoprotective effects of LPM likely depend on an Nrf2-dependent mechanism.

## 1. Introduction

In the past 20 years, a significant paradigm shift has relocated the idea of chronic illness etiology to the gut, where disease phenotypes may be contagious simply by transferring luminal material from a diseased to a healthy animal. The intestinal 3M (microbiome, metabolism and metabolome) interactions have emerged as a critical influencer of total human well-being independent of the genome–environment interaction [1]. Especially the gut metabolome, including diet-derived or microbial metabolites, is essential in controlling intestinal immunometabolic homeostasis. Gut metabolites are one of the major drivers of interkingdom interactions and can uphold mutualistic, commensal and pathogenic connections between the host and microbe. These metabolites can affect the host’s energy homeostasis, fat storage, glucose metabolism, immune balance and endocrine function by affecting extraintestinal tissue. The altered intestinal metabolome is often associated with noncommunicable chronic metabolic diseases such as diabetes and obesity [2].

Gut commensals, primarily the probiotic bacteria, are known to limit mucosal inflammation and attenuate the loss of gut-barrier function, preventing the portal translocation of gut-derived pyrogenic metabolites to the liver and the systemic circulation [3]. Although the increased diversity and abundance of probiotic bacteria are considered a microbial marker of health, how probiotic bacteria exert beneficial activities to the extraintestinal tissue remains critically underexplored. Since the enrichment of probiotic bacteria is associated with a functional gut barrier, paracellular translocation of probiotic bacteria to exert extraintestinal metabolic benefits seems unlikely. Such conditions would result in probiotic-associated septicemia and dire clinical consequences [4,5]. Moreover, since the intestinal mucosal layer is covered by a thick layer of mucin and remains populated by mucin-degrading commensals (e.g., *Akkermansia*), physical interaction between gut commensals and the enterocytes to exert immunometabolic homeostasis also seems to be unlikely. In contrast, gut microbial metabolites could relatively efficiently pass through the gut barrier, reach the portal circulation and affect hepatic immunometabolic homeostasis.

Supplementation of probiotic bacteria, or prebiotics, to enrich the populations of gut probiotics is an effective strategy for mitigating metabolic disease along the gut–liver axis. *Lactiplantibacillus plantarum* (formerly *Lactobacillus plantarum*) is a probiotic bacterium commercially exploited extensively. Earlier studies have demonstrated that oral supplementation of live *L. plantarum* in high-fat-diet-fed mice attenuates nonalcoholic steatohepatitis by altering the gut microbial profile and modulating hepatic fatty acid metabolic pathways [6]. Live *L. plantarum* also attenuates alcoholic liver injury by mitigating hepatic inflammation and elevating oxidative stress response in mice [7]. Heat-killed *L. plantarum* was demonstrated to attenuate oxidative damage-induced liver damage in association with improving gut-barrier function and reducing proinflammatory gene expression in rats [8]. Treatment of HepG2 cells with heat-inactivated *L. plantarum* resulted in protection from LPS/TLR4-dependent inflammatory injury by modulation of Toll-like receptor (TLR) negative regulators of mitogen-activated protein kinase and NF-κB-dependent signaling [9]. The supplementation of live *L. plantarum* holds promising potential to exert metabolic health benefits. However, since inactivated *L. plantarum* are not metabolically active, it is likely that the hepatoprotective activities of *L. plantarum* are independent of the cell viability. Therefore, to test this hypothesis, cell-free *L. plantarum* metabolites (LPM) were evaluated for their potential to protect against oxidative-stress-induced hepatic injury using HepG2 cells. Collectively, the data indicated that LPM holds the potential to attenuate oxidative-stress-induced hepatic damage, and the intracellular mechanism was explored.

## 2. Materials and Methods

### 2.1. Bacterial Strain

Pure culture of *L. plantarum* MTCC 2621 (AHIFPC/Equivalent to MTCC: ATCC8014, DSM20205, ICPB2080, NCDO82, NCIB63) was obtained from the Microbial Type Culture Collection (MTCC) (CSIR-Institute of Microbial Technology, Chandigarh, India) and cultured under standard conditions. These strains were maintained as frozen (−80 °C) stocks in MRS broth supplemented with 20% (*v*/*v*) glycerol. They were transferred at least three times consecutively using a 1% (*v*/*v*) inoculum in Man, Rogosa and Sharpe (MRS) broth at 37 °C for 18 h before use.

### 2.2. Evaluation of Probiotic Characteristics

The probiotic characteristics of *L. plantarum* were evaluated by studying its tolerance against various biotic factors as per standard guidelines [10]. In brief, bile acid tolerance was determined by growing *L. plantarum* in varying concentrations of bile acid infused in MRS broth (HiMedia, Thane, India). *L. plantarum* was initially grown overnight in MRS broth, then 10^6^ CFU/mL cell suspension was added to 20 mL freshly prepared MRS broth containing 0–1% bile acid. Temperature tolerance was studied by inoculating *L. plantarum* in MRS broth containing separate flasks that were incubated at 25–45 °C. For salt tolerance, *L. plantarum* was incubated in MRS broth with 0–4% concentration of NaCl. To evaluate pH tolerance, the pH of MRS broth was adjusted to 2–8 using HCl or NaOH, and *L. plantarum* was inoculated in separate flasks. All flasks were incubated for 24 h at 37 °C at 120 rpm.

### 2.3. Sample Preparation and Gas Chromatography–Mass Spectrometry (GC-MS) Analysis

*L. plantarum* (1 × 10^6^ CFU/mL initial count) was cultured in 500 mL MRS broth for 48 h under constant shaking and then centrifuged (5000 rpm for 10 min) to isolate the cell pellet. Cells were washed twice with PBS to remove residual components of the MRS media. The cell pellet was resuspended in chilled methanol:water (3:1, *v*/*v*) and vortexed vigorously for 15 min, then centrifuged at 10,000 rpm for 10 min at 4 °C. The supernatant was passed through a 0.4 μm filter to remove cells, and the filtrate was collected in a separate vial, dried under N_2_. The resultant was considered as LPM. LPM was mixed with 20 µL of *N*,*O*-bis (trimethylsilyl) trifluoroacetamide + trimethylchlorosilane (99:1 *v*/*v*) mixture and incubated for 60 min at 25 °C with occasional vortex and then sealed in autosampler vials with polytetrafluoroethylene cap using N_2_ flushing. Pooled metabolite extract (*n* = 3) was analyzed using Shimadzu QP 2010 Ultra GC-MS instrument equipped with a TG-5MS column (30 m × 0.25 mm × 0.25 µm). The injector temperature was set at 250 °C, and the initial temperature of the program was set at 60 °C (solvent delay 4 min) with a hold of 4 min, followed by a ramp of 10 °C to 300 °C with a hold of 10 min. Derivatized samples (1 µL) were injected in a split mode (split ratio 20:1) with a splitless time of 0.80 min, with a constant flow of helium gas (1 mL/min). MS transfer line temperature was set at 290 °C with an ion-source temperature of 200 °C (electron ionization). The samples were analyzed at electron energy 70 eV (vacuum pressure: 22.21 × 10^−0.5^ Torr), and the mass analyzer range was set to 50–650 amu. MS data were analyzed using Automated Mass Spectral Deconvolution and Identification System (AMDIS) version 2.70. The major and essential compounds were identified by mass fragmentation patterns (*m*/*z*) of the reference parent compound (molecular peak and base peak) using MS Interpreter version 2.0 and by matching with the reference database of the National Institute Standard and Technology (NIST) with an MS Library V2011.

### 2.4. Enrichment and Pathway Analysis

MetaboAnalyst V5 was used to analyze the biochemical pathway enrichment using the metabolite abundance data sets obtained from the GC-MS analysis [11]. Enrichment analysis was performed based on the Kyoto Encyclopedia of Genes and Genomes (KEGG) and was used to investigate how groups of functionally related metabolites are significantly enriched that would potentially eliminate requirements of preselect compounds based on arbitrary cut-off thresholds. Identified metabolites were mapped against PubChem and KEGG identifiers. Pathway analysis was performed based on KEGG identifiers, where out-degree centrality was used for topology analysis, and Fisher’s Exact Test was used as enrichment method.

### 2.5. Antioxidant and Free-Radical Scavenging Assays

Based on the intracellular free-radical forming mechanisms, a total of multiple in vitro free radical scavenging assays (i.e., hydroxyl radical, OH^•^; superoxide radical, O_2_^•–^; singlet oxygen, ^1^O_2_; hypochlorous acid, HOCl; hydrogen peroxide, H_2_O_2_; nitric oxide, NO; peroxynitrite, OONO^–^) were selected to assess the overall antioxidant activities of LPM [12]. The reducing potential of LPM as a surrogate indicator of the general antioxidant activity was measured using 2,2-diphenyl-1-picrylhydrazyl (DPPH) assay. Since transition metals can accelerate intracellular free-radical formation cascade by potentiating the Fenton reaction, iron-chelation activity was measured, and to evaluate the potential of LPM to limit free-radical mediated peroxidation of cellular lipids, a lipid peroxidation assay was performed using a chicken brain sample obtained from a local slaughterhouse. All assays were performed against appropriate standards per previously standardized methods adapted to a reduced volume suitable for microplates [13,14,15]. The range of the highest sample dose for each assay was based on the linear response range for respective standard compounds in the final volume of the reaction mixture.

### 2.6. HepG2 Cell Culture

The human hepatocarcinoma HepG2 cell line obtained from National Center for Cell Science (India) was maintained in low-glucose Dulbecco’s Modified Eagle Medium (HiMedia, India) supplemented with 10% fetal bovine serum, 100 UI/mL penicillin, 100 μg/mL streptomycin and 25 μg/mL amphotericin B. Cells were grown at 37 °C and 5% CO_2_ under controlled humidity. Sub-culturing was performed at approximately 45–50 h intervals, and cell growth was monitored with an Olympus inverted microscope. Cell count was performed by trypan blue method using an automated cell counting system (Farscope B, Curiosis, Seoul, Republic of Kore).

### 2.7. MTT Cell Viability Assay

Microwell-plate-based 3-(4,5-dimethylthiazol-2-yl)-2,5-diphenyl-2H-tetrazolium bromide (MTT) cell viability assay was performed to evaluate the dose-and time-dependent effects of LPM on the viability of HepG2 cells [16]. In brief, HepG2 cell suspension was prepared (1 × 10^6^ cells/mL) in DMEM, and 100 μL was seeded for 24 h to achieve cell adhesion and 80% confluency. Then, LPM (in DMEM) was added to appropriate wells to achieve a final concentration of 0–10 μg/mL. The plate was then incubated for 24 h under standard conditions. After the incubation period, 20 mL of MTT solution (5 μg/mL, dissolved in DMEM; pH 7.0) was added to each well, the plate was covered with aluminum foil and incubated for 4 h at 37 °C in a humidified incubator. After incubation, 150 μL of the suspension from each well was taken out without disturbing the bottom layer, and 150 μL of DMSO was added to each well and mixed thoroughly. Finally, the optical density (O.D.) was taken at 540 nm (ThermoScientific, Waltham, MA, USA). Cells were separately cultured for 0–24 h under the treatment of 2.5 μg/mL LPM to evaluate the time-dependent effects of LPM on cell viability.

### 2.8. In Vitro Experimental Design

To study the intracellular cytoprotective mechanism of LPM, 2 × 10^6^ HepG2 cells were seeded in 6 well plates for 30 h to reach 75% confluency. Next, cells were treated with 200 μM treatment dose-selection of H_2_O_2_ was based on prior report demonstrating the dose-dependent free radical formation and corresponding cell injury by H_2_O_2_ treatment of HepG2 cells beyond 400 μM [17]. H_2_O_2_-treated cells were separately supplemented with LPM at 2.5 μg/mL prepared in DMEM. In a separate set of experiments, all cells were pre-treated with 5 μM of ML385 (Sigma-Aldrich, St. Louis, MO, USA, SML1833), an inhibitor of nuclear factor erythroid 2-related factor 2 (Nrf2) for 12 h, and then subsequently treated with H_2_O_2_ alone or in conjuncture with LPM. After incubation for 24 h, cell culture supernatant and cells were collected separately. LDH leakage assay was performed to measure membrane damage as a surrogate marker of cell viability using a commercial kit (Tulip Diagnostics, Amritsar, India).

### 2.9. Biochemical Assays

Cells, after appropriate treatment (Section 2.8), were washed in PBS, harvested in chilled cell lysis buffer and divided into multiple aliquots to be used for the biochemical assays of antioxidant enzymes. Peroxidase activity was measured using the cell lysate spectrophotometrically at 436 nm by measuring the oxidation of guaiacol as per established method [18]. The catalase activity was measured by monitoring the breakdown of H_2_O_2_ by cellular catalase at 240 nm following standard procedure (Luck, 1965). GSH was measured using an ELISA kit according to the manufacturer’s protocol (LifeSpan BioSciences, Shirley, NY, USA) and normalized to the total cellular protein content.

### 2.10. Nitric Oxide Release Assay

The amount of nitric oxide (NO) released in the culture supernatant was measured following the Griess reagent method [19]. Briefly, 100 μL of the culture supernatant was mixed with 400 μL of Griess reagent (1% sulfanilamide and 0.1% *N*-(1-naphthyl) ethylenediamine hydrochloride in 2.5% H_3_PO_4_) in a 96-well plate. The plate was incubated for 20 min at room temperature, and the generated purple azo-dye formed was detected at 540 nm in a microwell plate reader (Thermo Scientific multi-scan spectrum). Data expressed as a percent of inhibition relative to untreated control.

### 2.11. Gene Expression Studies

A gene expression study was performed following our previous method [20,21]. In brief, after appropriate treatment, cells were centrifuged at 5000× *g* for 10 min to separate from the culture media. The cell pellet was washed twice with ice-cold PBS to remove residual media components. Total mRNA from the HepG2 cells was extracted using TRIZOL reagent (ThermoFisher Scientific, Waltham, MA, USA) method according to the manufacturer’s instructions, and quantification was performed in nanodrop (Thermo Scientific, Waltham, MA, USA). cDNA was synthesized using an iScript reverse transcription kit, and gene expression studies were performed using SYBR green PCR kit on a Real-Time instrument (Bio-Rad, CFX96). Primers for tumor necrosis factor-α (TNF-α; forward AGCCCATGTTGTAGCAAACC, reverse GGAAGACCCCTCCCAGATAG), interleukine-6, (IL-6, forward TTCCACGAAGTGACAGTGTGA, reverse GCACGGTAGAAAAGGAAGGGT) and 18S (forward CGCTTCCTTACCTGGTTGAT; reverse GAGCGACCAAAGGAACCATA) were procured from Sigma-Aldrich. Target genes were quantified relative to 18S using the 2^−ΔΔCT^ method.

### 2.12. Statistical Analysis

All quantitative data are reported as the mean ± SEM of 3–6 measurements. Two-way RM ANOVA was performed to assess the effects of individual variables (e.g., temperature, pH, etc.) and time and their interactions using GraphPad (Boston, USA) V8. Tukey’s post hoc test was utilized to calculate the differences in AUC_0–24h_ for individual treatments. For in vitro antioxidant assays, statistical analysis was performed by unpaired *t*-test. The percentage of inhibition/scavenging was calculated by the formula $\frac{X0-X1}{X0}\times 100$, where *X*0 was the absorbance of the control, and *X*1 was the absorbance in the presence of the samples and standard. Statistical group-comparisons for HepG2 cell experiments were performed using one-way ANOVA followed by Tukey’s test. The enrichment ratio for metabolites and pathways was calculated based on observed hit/expected hit values. Holm–Bonferroni correction for *p*-value and false discovery rate (FDR) was calculated for all entries of enrichment analysis. Pathway analysis was performed using Fisher’s exact test. *p* < 0.05 was considered significant for all cases.

## 3. Results

### 3.1. Growth Parameters

**Figure 1:**
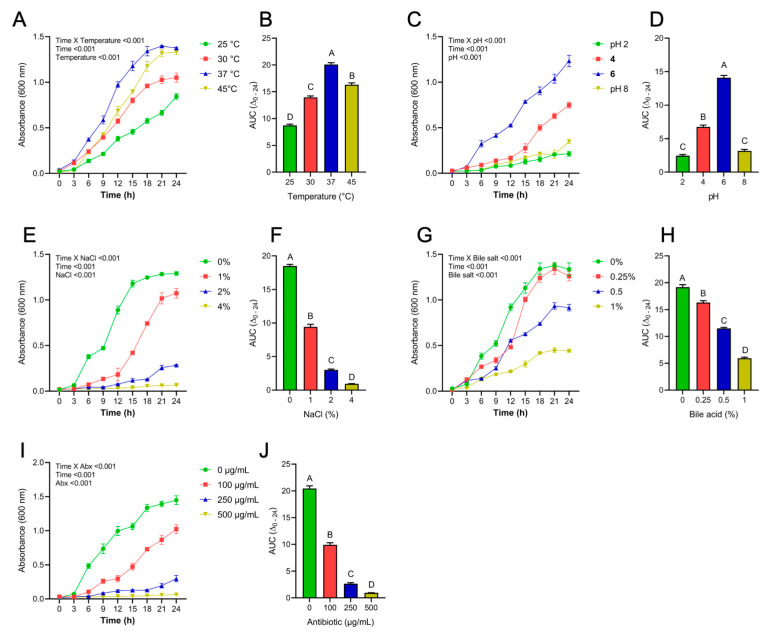
Growth curves and corresponding area under the curve (AUC) of *L. plantarum* grown under different stress conditions. The stress conditions include (**A**,**B**) temperature; (**C**,**D**) pH; (**E**,**F**) sodium chloride; (**G**,**H**) bile salt; and (**I**,**J**) antibiotic ampicillin. Data represented as mean ± SEM of 3 measurements. Data analysis was performed by 2-way repeated measure ANOVA to evaluate the effects of individual variables and their interaction with time. Tukey’s post hoc test was used to calculate the differences in AUC_0_–_24h_ for each treatment. Groups not sharing a common letter are significantly different (*p* ≤ 0.05).

The biotic and abiotic stress tolerance potentials of LP were measured by growing LP under variable conditions of bile acid, temperature, NaCl, temperature and antibiotic (ampicillin). For temperature (Figure 1A,B), optimum growth was observed at 37 °C followed by 45, 30 and 25 °C. The growth kinetics of LP was optimum at pH 6, which is closer to the normal pH of MRS broth (pH 6.5), followed by pH 4 and 2 (Figure 1C,D). In the case of NaCl tolerance, a dose-dependent decrease in growth rate was observed, with a sharp 49% decrease in AUC at 1% NaCl concentration compared to 0% NaCl (Figure 1E,F). For bile acid tolerance (Figure 1G,H), data demonstrated a dose-dependent decreased growth potential of LP with optimum growth under 0% bile. Although the growth rate was lower, LP was able to tolerate bile acid at 1% concertation. LP demonstrated better growth kinetics without antibiotic treatment, which was affected in a dose-dependent manner with a 51.6% decrease in AUC at 100 μg/mL concentration (Figure 1I,J). Bi-factorial ANOVA indicated significant (*p* < 0.01) independent effects of time and all the stress parameters and significant interactive effects of time vs. stress factors on bacterial growth patterns.

### 3.2. Metabolomic Fingerprinting of LPM

**Figure 2:**
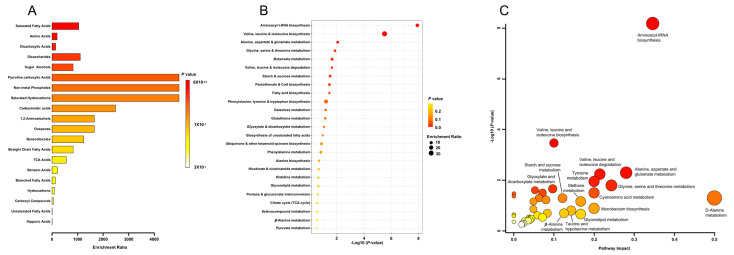
Metabolite sub-class enrichment analysis (**A**), metabolic pathway enrichment (**B**) and metabolic pathway impact analysis (**C**) of *L. plantarum* metabolites (LPM). Untargeted chemical fingerprinting of LPM was performed using GC-MS after sample derivatization using silylation. Identified compounds are enlisted in Supplementary Table S1. The compounds and corresponding abundance scores were utilized in MetaboAnalyst 5.0 to identify the chemical class and pathway enrichments. Relevant additional data are provided in the supplementary document.

A total of 66 metabolites were identified in LPM (Supplementary Table S1), of which the top 10 predominant metabolites were glycerol, 5-oxoproline, l-proline, sucrose, hexadecanoic acid, l-threonine, *N*,*N*-dimethylglycine, ethyl *N*,*N*-diethylcarbamate, l-valine and butanedioic acid (Supplementary Table S1). However, upon chemical classification, it was revealed that saturated fatty acids, amino acids, dicarboxylic acids, disaccharides and sugar alcohols were the most significantly enriched chemical class, while pyrroline carboxylic acids, non-metal phosphates, saturated hydrocarbons, carboximidic acids and 1,2-aminoalcohols had the highest enrichment ratios (Figure 2A and Supplementary Table S2). Enrichment analysis of microbial biosynthetic pathways based on the identified metabolites revealed that aminoacyl-tRNA biosynthesis; valine, leucine and isoleucine biosynthesis; alanine, aspartate and glutamate metabolism; glycine, serine and threonine metabolism; and butanoate metabolism were most significantly enriched (Figure 2B and Supplementary Table S3). Metabolic pathway impact analysis revealed that novobiocin biosynthesis; D-alanine metabolism; aminoacyl-tRNA biosynthesis; alanine, aspartate and glutamate metabolism; and glycine, serine and threonine metabolism had the highest impact on the metabolite diversity in LPM (Figure 2C and Supplementary Table S4).

### 3.3. Antioxidant Test

**Figure 3:**
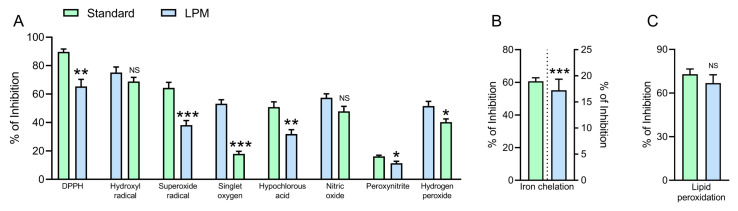
Results of in vitro antioxidant and free-radical scavenging activities of *L. plantarum* metabolites (LPM). LPM at 100 μg/mL was compared with the equal concentration of respective assay standards. Standards used in specific assays were ascorbic acid (DPPH assay and hypochlorous acid), mannitol (hydroxyl radical scavenging), quercetin (superoxide anion), lipoic acid (singlet oxygen scavenging), curcumin (nitric oxide), gallic acid (peroxynitrite scavenging), sodium pyruvate (hydrogen peroxide), ethylenediaminetetraacetic acid (iron chelation activity) and Trolox (lipid peroxidation assay). Data show (**A**) general antioxidant assays, (**B**) Iron chelation assay, (**C**) Lipid peroxidation assay. Data analysis was performed using 2-tailed unpaired *t*-tests. Data represented as mean ± SEM of 6 measurements. * *p* < 0.05, ** *p* < 0.01, *** *p* < 0.001 and ^NS^ *p* > 0.05.

All antioxidant tests were performed at 100 μg/mL concentrations of LPM and respective assay standards. LPM demonstrated comparable (*p* > 0.05) activities with the respective standards for hydroxyl radical and nitric oxide scavenging, whereas, for all other assays, LPM demonstrated significantly lower (*p* < 0.05) bioactivities relative to the equal concentrations of assay standards (Figure 3). Compared to the respective assay standards, the free-radical scavenging activities of LPM were lower at 27% for DPPH, 40.7% for superoxide radical, 66.3% for singlet oxygen, 37.2% for HOCl, 29.8% for peroxynitrite and 21.9% for H_2_O_2_ scavenging. At a concentration of 100 μg/mL, LPM demonstrated 71.6% lower iron chelation activity compared to standard EDTA at 2 μg/mL (Figure 3B). LPM demonstrated 29.8% lower lipid peroxidation relative to the standard Trolox.

### 3.4. Cell Viability

**Figure 4:**
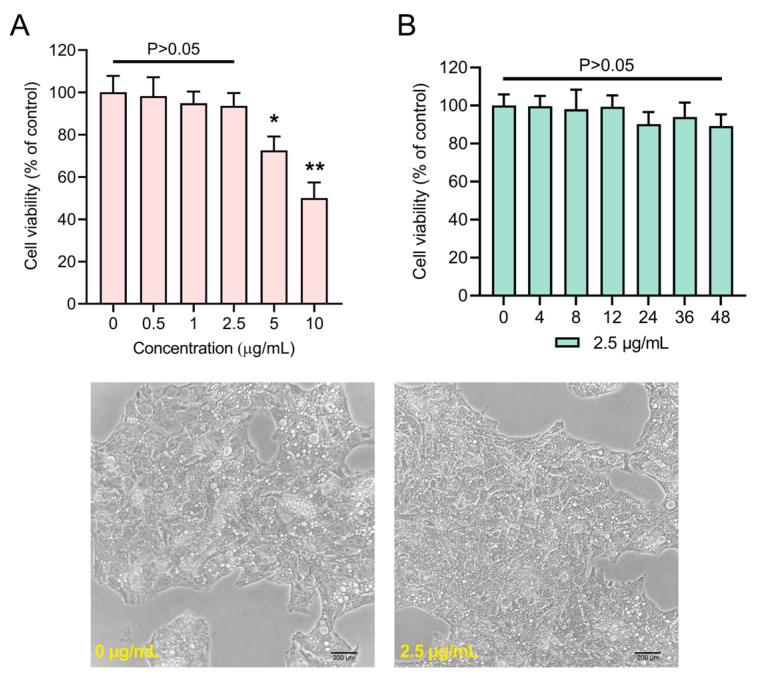
Cytotoxic effects of *L. plantarum* metabolites (LPM) in (**A**) dose- and (**B**) time-dependent manners. LPM was dissolved in cell culture media and treated against HepG2 cells. Lower panels represent HepG2 cells treated with or without LPM. Data represented as mean ± SEM of 6 measurements. * *p* < 0.05 and ** *p* < 0.01.

An MTT cell viability assay was performed to identify the treatment dose of LPM for HepG2 cells. Data showed a significant loss of viability of HepG2 cells at 5 μg/mL (72.6% viability) and 10 μg/mL (50.1% viability) concentration compared to control (Figure 4). Although 6.4% loss of viability was observed at 2.5 μg/mL concentration, the drop in viability was not significant (*p* > 0.05) compared to the control. Therefore, the sub-lethal 2.5 μg/mL concentration of LPM was chosen for subsequent experiments. Further, we wanted to verify the nonlethal effects of LPM by recording the temporal effects on cell viability. Data showed that treatment of HepG2 cells with LPM at 2.5 μg/mL for 48 h had a dose-dependent loss of viability response, but the loss of viability was insignificant (10.8%, *p* > 0.05).

### 3.5. LPM Exerts Cytoprotective Effects against H_2_O_2_-Induced Injury

**Figure 5:**
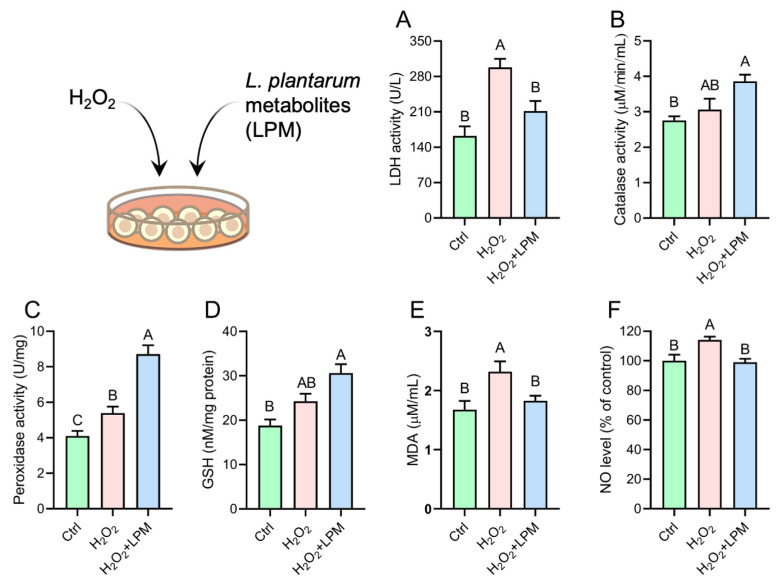
Effects of *L. plantarum* metabolites (LPM) on the (**A**) lactate dehydrogenase (LDH) leakage, (**B**) catalase activity; (**C**) peroxidase activity; (**D**) glutathione level; (**E**) malondialdehyde (MDA) release; and (**F**) nitric oxide (NO) release in HepG2 cells that were treated with hydrogen peroxide (H_2_O_2_). Data represented as mean ± SEM of 3 measurements. Groups not sharing a common letter are significantly different (*p* ≤ 0.05).

To evaluate the cytoprotective potentials of LPM, HepG2 cells were treated with H_2_O_2_ for oxidative cellular damage. The extent of cell damage was assessed by measuring LDH leakage from the cells into the culture medium. Data showed that H_2_O_2_ resulted in 83.6% increased LDH release compared to control, which was lowered by 29% due to LPM treatment (Figure 5A). Next, the activities of various intracellular antioxidant enzymes were measured in response to the treatment. Data showed that H_2_O_2_ treatment increased cytoprotective enzymatic activities in the range of 11.3–31.3% (Figure 5B–D). However, compared to the control, only the level of peroxidase activity was significantly elevated in response to H_2_O_2_. LPM treatment on H_2_O_2_-treated cells resulted in 40.4–112.1% higher levels of the antioxidant enzymes, out of which only the increase in peroxidase (61.5%) was significant compared to the cells that were treated with H_2_O_2_ alone. The levels of all the antioxidant enzyme activities were significantly higher in the LPM-treated cells compared to the controls. The level of lipid peroxidation, as indicated by the measurement of MDA, was 38.3% higher in the H_2_O_2_-treated cell. The level of H_2_O_2_-induced MDA was 21.2% lower when the cells were treated with LPM. The level of NO release in the culture supernatant was increased by 14.2% due to H_2_O_2_ treatment, which was reduced by LPM to the level not different from the control cells.

### 3.6. Cytoprotective Effects of LPM Were Diminished upon the Inhibition of Nrf2

**Figure 6:**
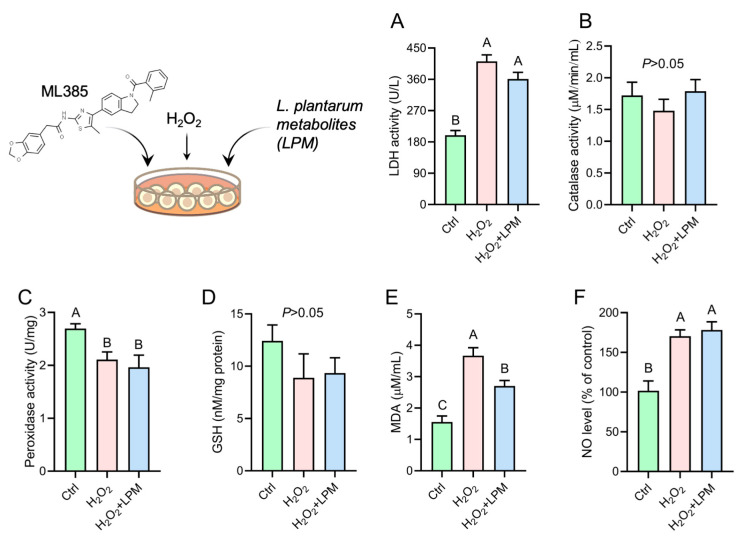
Effects of *L. plantarum* metabolites (LPM) on the (**A**) lactate dehydrogenase (LDH) leakage; (**B**) catalase activity; (**C**) peroxidase activity; (**D**) glutathione level; (**E**) malondialdehyde (MDA) release; and (**F**) nitric oxide (NO) release in HepG2 cell that was treated with hydrogen peroxide (H_2_O_2_) and pretreated with ML385, a pharmacological inhibitor of nuclear factor erythroid 2–related factor 2. Data represented as mean ± SEM of 3 measurements. Groups not sharing a common letter are significantly different (*p* ≤ 0.05).

Since Nrf2 plays a central role in cytoprotection against free-radical-mediated injury, we wanted to understand whether the cytoprotective effects of LPM were dependent on Nrf2-dependent mechanisms. For this, HepG2 cells were treated with purified ML385, a pharmacological inhibitor of Nrf2-dependent downstream signaling. Data showed that H_2_O_2_ treatment resulted in 2.1-times increased LDH release compared to the control cells (Figure 6A). Although LPM treatment lowered the LDH leakage to 12.2%, the reduction was insignificant (*p* > 0.05). In the case of catalase and GSH, the activities of both the cytoprotective enzymes were statistically unaffected by the H_2_O_2_ and LPM treatments (Figure 6B,D). However, compared to the control, H_2_O_2_ in ML385-treated cells resulted in a significant 21.9% reduction in the peroxidase activities, which LPM could not attenuate. In line, H_2_O_2_ treatment also resulted in a 2.7-times increased MDA level. Although LPM reduced the MDA level by 26.2%, it was significantly higher than that of controls. The level of NO in the cell culture media was 1.7-times increased in response to H_2_O_2_, which remained unchanged when cells were treated with LPM.

### 3.7. LPM-Mediated Attenuation of H_2_O_2_-Dependent Inflammation Is Partially Mediated Nrf2-Dependent Mechanism

**Figure 7:**
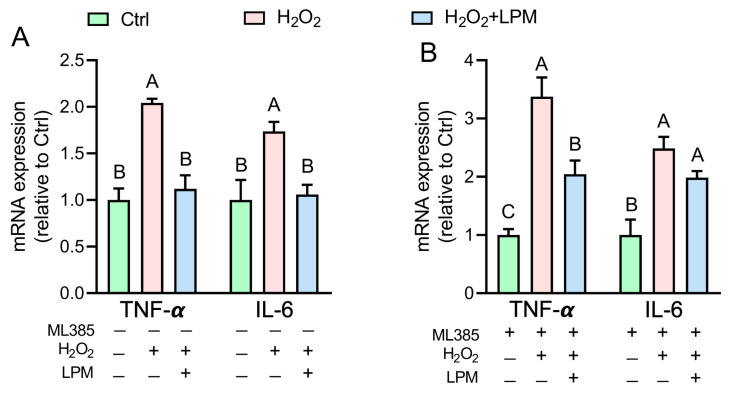
Effects of *L. plantarum* metabolites (LPM) on the mRNA expression of tumor necrosis factor-α (TNF-α) and interleukin-6 (IL-6) in H_2_O_2_-treated HepG2 cells without (**A**) or with (**B**) pretreatment of ML385. Data represented as mean ± SEM of 3 measurements. Groups not sharing a common letter are significantly different (*p* ≤ 0.05).

In line with previous reports that oxidative stress evoked due to H_2_O_2_ treatment could elicit an inflammatory response [22], we endeavored to investigate the effects of LPM testament on H_2_O_2_-induced inflammation. Data showed that control to control, H_2_O_2_ treatment resulted in significantly increased mRNA expression of TNF-α (2.04 times) and IL-6 (1.73 times) (Figure 7A). LPM treatment attenuated the mRNA expression of TNF-α (45.1%) and IL-6 (38.7%) to levels no different from the controls. However, H_2_O_2_ treatment in ML385 pretreated cells resulted in significantly higher mRNA expressions of TNF-α (3.37 times) and IL-6 (2.48 times) (Figure 7B). LPM treatment although significantly lowered the mRNA expression of TNF-α (39.4%), the attenuation of IL-6 mRNA expression was not to the level observed in controls.

## 4. Discussion

The findings of this study demonstrate that, in agreement with our hypothesis, cell-free metabolites of LPM can exert cytoprotection against H_2_O_2_-induced oxidative stress. The most significantly enriched classes of metabolites in LPM were saturated fatty acids, amino acids and dicarboxylic acids, which were associated with the scavenging of free radicals in vitro. LPM treatment in H_2_O_2_-treated HepG2 cells improved the activities of intracellular antioxidant enzymes in association with the attenuation of lipid peroxidation and the mRNA expressions of proinflammatory genes. However, the cytoprotective activities of LPM were diminished upon the pharmacological inhibition of Nrf2. These data collectively indicate that the probiotic activities of *L. plantarum* at the extraintestinal tissue could be exerted by LPM independent of viable cells. However, the cytoprotective activities of LPM are likely governed by Nrf2-dependent mechanism.

The probiotic potential of viable *L. plantarum* supplementation has been demonstrated utilizing various experimental models. In rats with diet-induced NAFLD, 5-week treatment with *L. plantarum* NCU116 lowered oxidative stress and improved liver function while reducing hepatic fat buildup [23]. Specifically, *L. plantarum* favorably modulated the hepatic lipid metabolism while lowering the levels of endotoxin and proinflammatory cytokines in the liver. *L. plantarum* HFY09 mitigates alcoholic liver injury by upregulating the levels of superoxide dismutase and glutathione and attenuating the expressions of IL-6, IL-1β, TNF-α [7]. The metabolic health-beneficial effects of viable probiotic cell supplementation are likely associated with increased gastrointestinal colonization of the probiotic bacteria, promotion of the commensal population and inhibition of outgrowth of the pathobionts [1]. However, animal studies have shown that inactivated probiotic bacteria also hold the potential to mitigate experimental metabolic insults [24]. Indeed, clinical studies investigating inactivated *L. plantarum* are critically lacking, but a randomized, controlled trial demonstrated that daily supplementation of heat-inactivated *L. plantarum* L-137 reduced inflammation and improved lipid metabolism in 100 Japanese subjects [25]. Specifically, heat-killed *L. plantarum* supplementation for 12 weeks reduced the levels of aminotransferases, LDL-cholesterol and C-reactive protein in the subjects. This evidence collectively shows that inactivated probiotic bacteria are likely equally effective in mitigating metabolic disease, indicating the prophylactic potentials of the metabolites from probiotic bacteria. If this is true, clinical cases of opportunistic infections arising from unprescribed probiotic consumption could be circumvented while achieving disease prophylaxis [4,5].

An earlier study demonstrated that treatment of *L. plantarum* culture supernatant limits hepatocellular lipid accumulation by downregulating the mRNA expressions of lipid-metabolism-related genes, including acetyl-CoA carboxylase, fatty acid synthase, sterol regulatory element-binding protein 1 and peroxisome-proliferator-activated receptor-γ [26]. However, the composition of the *L. plantarum* global metabolome remained unexplored. In the present study, we identified the optimum growth parameter of *L. plantarum* against several biotic and abiotic stress conditions, grew the bacteria under such conditions and utilized the cell-free metabolome for cell-based assays. Among the highly abundant metabolites, the antioxidant effects of 5-oxoproline (aka pyroglutamic acid) [27], butanedioic acid (i.e., succinic acid) [28], *N*,*N*-dimethylglycine [29], etc. have already been established. Nevertheless, the collective antioxidant effects demonstrated by LPM were likely attributed to the additive free-radical scavenging activities of multiple metabolites since the majority of the metabolites have been detected in other lactic acid bacteria as well [30,31]. Further, it is also plausible that *L. plantarum* grown under altered conditions would produce a different array of metabolites contributing to altered bioactivities, but it remains out of the scope of the current study.

Prior studies have reported the antioxidant and free radical neutralizing activities of *L. plantarum* that contributed towards attenuation of signs of aging [32,33], dietary oxidation-induced liver injury [34,35], oxidative intestinal damage [36], improved myocardial diastolic function through antioxidant effects [37] and potential to improve the antioxidant properties of dietary supplements [38,39]. Although the intracellular mechanisms through which *L. plantarum* mitigate oxidative insult have been explored, whether cell-free metabolites of *L. plantarum* possess similar benefits remained unexplored. Since intestinal mucosal injury through the oxidative route leads to gut-barrier dysfunction facilitating translocation of gut microbial pyrogenic metabolites along the gut–liver axis [1,40], mitigating oxidative damage by scavenging reactive free-radicals would indirectly exert hepatoprotection by attenuating the loss of gut barrier. We evaluated the free-radical scavenging effects of LPM against the majority of intracellular reactive species. Although the bioactivities of LPM compared to the same concentrations of respective assay standards were lower for certain free-radicals, it is likely that *L. plantarum* grown under different growth conditions would generate altered metabolites which likely would result in altered bioactivities. Interestingly, LPM differentially affected the individual components of the Haber–Weiss reaction. The Fenton chemistry, as a part of the Haber–Weiss reactions, is considered a key feature of intracellular free-radical formation cascade where the transient but extremely reactive OH^•^ is produced from H_2_O_2_ in the presence of Fe^2+^ and later gets protonated to generate water [12,13]. Although LPM could not scavenge H_2_O_2_ or transform Fe^3+^ to Fe^2+^ to a level similar to the assay standards, the OH^•^ scavenging effects of LPM relative to standard mannitol were comparable. This indicates that LPM holds the potential to neutralize OH^•^ directly by protonating OH^•^, a feature essential for limiting the peroxidation of the plasma membrane. Indeed, the assay for lipid peroxidation utilizing OH^•^ as a peroxidation agent demonstrated comparable activities of LMP and the assay standard vitamin E analog Trolox. Collectively, these data demonstrated the potent non-enzymatic antioxidant effects of LPM, which could be instrumental in alleviating redox injury by scavenging reactive free radicals.

Since LPM can effectively neutralize OH^•^ generated by H_2_O_2_ in addition to scavenging H_2_O_2_, we intended to utilize an in vitro experimental model of H_2_O_2_-induced cellular injury to HepG2 cells. Controlled H_2_O_2_ treatment in cultured cells is known to cause membrane damage and exert apoptosis by eliciting intracellular free radical formation [41,42]. Specifically, H_2_O_2_ treatment can function as a double-edged sword, causing the loss of intracellular antioxidant defense and accumulation of reactive species resulting in cellular damage [43]. Additionally, intracellular oxidative damage has also been associated with chronic progressive liver disease [44], and oxidative injury is routinely utilized as an artificial experimental model to mimic the mode of in vivo disease pathogenesis [45]. In line with the in vitro data, it was observed that LPM could attenuate H_2_O_2_-induced cell injury to HepG2 cells. H_2_O_2_ can damage the plasma membrane, resulting in the loss of membrane integrity of various cells and causing leakage of LDH to the culture media [46,47,48]. LPM treatment resulted in the attenuation of the LDH levels. These data indicate that LPM can exert cytoprotection by neutralizing H_2_O_2_-induced membrane damage in HepG2 cells. One of the key consequences of H_2_O_2_-induced cytotoxicity is the peroxidation of membrane lipids. Indeed, increased production of MDA, the lipid peroxidation byproduct, has also been reported in NAFLD patients [49]. Earlier studies have shown that H_2_O_2_ treatment results in membrane lipid peroxidation and increased release of MDA from HepG2 cells [22]. In support, our data showed an increased level of MDA in the culture supernatant due to H_2_O_2_ that was attenuated to a level no different from the controls. These data collectively indicate that LPM can attenuate oxidative injury to the HepG2 cells and limit the peroxidation of membrane lipids. Although prior studies have demonstrated similar anti-lipid peroxidation effects of *L. plantarum* fermented dietary supplements on Caco-2 cells and heat-killed *L. plantarum* on HepG2 cells, this is the first demonstration of similar benefits of cell-free LPM on HepG2 cells.

In line with the in vitro free-radical scavenging effects of LPM and its anti-lipid peroxidation effects on HepG2 cells, we wanted to define whether these benefits are due to the additive free-radical scavenging effects of the metabolites or whether LPM can elicit intracellular cytoprotective mechanisms. Our data showed that H_2_O_2_-treated HepG2 cells resulted in increased activities of antioxidant enzymes with significant elevation in the peroxidase level. Although these observations remain in contrast to prior in vivo studies demonstrating that chemical-induced hepatotoxin that elicits free-radical-induced liver damage [50,51] and in vitro studies demonstrating diminished levels of antioxidant enzymes under redox stress, it is likely that elevated levels of antioxidant enzymatic activities occurred as a response to the H_2_O_2_-induced redox stress. Indeed, a previous study in leptin-resistant *db/db* mice with prominent oxidative hepatic injury demonstrated higher activation of intracellular cytoprotective machinery relative to normal controls [21]. Nevertheless, these changes at the cellular level are primarily dictated by the dose and duration of H_2_O_2_ treatment that can differentially affect the antioxidant enzymatic activities [52]. Treatment of H_2_O_2_-treated cells with LPM significantly increased the antioxidant enzymatic activities relative to non-treated controls. These data collectively demonstrated that LPM could provide cytoprotection against free-radical-induced cellular damage by upregulating the intracellular cytoprotective enzymes.

Next, we intended to understand whether the intracellular cytoprotective mechanisms of LPM depend on transcriptional regulation of the intracellular pathway. For this purpose, we pre-treated the cells with ML385, a pharmacological inhibitor of the transcription factor nrf2 [53]. The Nrf2-dependnet antioxidant response element pathway plays a critical role in regulating intracellular anti-oxidative responses and protecting cellular components from electrophile and oxidative damage [54]. Upon cellular exposure to oxidants, Nrf2 can transcriptionally activate the expressions of antioxidant response enzymes. Upon pretreatment with ML385, the cells were more affected by H_2_O_2_ treatment than non-ML385 treated cells. Specifically, H_2_O_2_ treatment significantly increased the LDH release and MDA levels while the antioxidant enzyme activities remained diminished. The peroxidase activity was significantly reduced due to ML385 treatment. LPM treatment neither protected from membrane damage nor improved the levels of antioxidant enzymes. The phenomenon that the cytoprotective effects of LPM on H_2_O_2_-treated cells were not observed in ML385-treated cells indicated that LPM activities depend on Nrf2, where pharmacological inhibition of Nrf2 diminish the cytoprotective benefits of LPM. In support, earlier studies have reported that probiotic *L. plantarum* can improve myocardial diastolic function [37] and exert antioxidant and hypolipidemic activities [55] in an Nrf2-dependent manner. However, the cytoprotective effects of LPM are likely not completely dependent on Nrf2 since LPM could significantly reduce the MDA but not to the level observed in the controls. Indeed, this was supported by a prior study demonstrating that 5-methoxyindoleacetic acid, a metabolite from *Lactobacillus* spp., can exert hepatoprotective benefits by activation of Nrf2-dependent antioxidant defense.

In line with earlier reports that oxidative cellular injury triggers a proinflammatory response in the hepatocytes [56] and that oxidative stress is associated with chronic inflammatory liver diseases [57], we intended to evaluate whether LPM treatment that improved antioxidant response would also mitigate H_2_O_2_-induced inflammation. Data showed that LPM attenuated the mRNA expressions of TNF-α and IL-6 that were elevated due to H_2_O_2_ treatment. This is important since not only that TNF-α and IL-6 are two of the key pro-inflammatory mediators under the transcription regulation of NFκB and STAT3, respectively, that are associated with chronic liver injury, and both of these transcription factors are also redox sensitive [58,59]. However, pretreatment of cells with ML385 diminished the anti-inflammatory effects of LPM to a certain extent, indicating an Nrf2-dependent anti-inflammatory activity of LPM. Indeed, Nrf2 is known to affect inflammatory response by transcriptionally regulating the level of heme oxygenase-1 and is a therapeutic target for chronic inflammatory diseases [60]. Indeed, our findings remain in parallel to prior findings demonstrating that *L. plantarum* protects against TLR4-induced inflammation in HepG2 cells by modulating Toll-like receptor negative regulators of MAP kinase and NF-κB signaling [9].

## 5. Conclusions

Results from this study collectively indicate that chemically defined cell-free metabolites of *L. plantarum* can attenuate H_2_O_2_-induced oxidative stress-mediated injury to HepG2 cells, likely in an Nrf2-dependent mechanism. Under in vivo experimental models with *L. plantarum* supplementation as a probiotic, the hepatoprotective effects observed are likely due to the translocation of bacterial metabolites along the gut–liver axis. This is not only supported by the targeted hepatoprotective effects demonstrated in the current set of data but also due to the fact that viable probiotic supplementation would strengthen the gut barrier and limit bacterial translocation to the liver. Therefore, only bacterial metabolites would be small enough for paracellular translocation and exert metabolic health benefits along the gut–liver axis. It is further possible that LPM can limit inflammatory and oxidative injury to the mucosal epithelium, resulting in improved gut-barrier function; however, this remains unexplored. In line with the use of probiotics for metabolic health benefits while limiting opportunistic infections caused by the probiotic bacterial overdose, metabolites from probiotic bacteria (LPM, for instance) would be preferable as a prophylactic strategy. However, it is to be noted that the diversity of metabolites produced by bacteria depends on multiple factors such as growth conditions, growth phase and availability of nutrients. Changes in the metabolites is expected to influence the bioactivities. In the present study, the bioactivities of LPM and its composition was dependent on the factors under which LPM was cultured. Therefore, future studies are required to obtain LPM with different metabolite signatures that would likely demonstrate cytoprotection and diverse bioactivities along the gut–liver axis.

## Data Availability

The datasets analyzed during the current study are available from the corresponding author on reasonable request.

## Supplementary Materials

The following supporting information can be downloaded at: https://www.mdpi.com/article/10.3390/antiox12040930/s1, Table S1: Chemical composition of cell-free *L. plantarum* metabolites. Table S2: Chemical sub-class enrichment data of cell-free *L. plantarum* metabolites. Data corresponds to Figure 2A of the manuscript. Table S3: Represents KEGG pathway enrichments based on the metabolites identified in the cell-free *L. plantarum* metabolites. Data corresponds to Figure 2B of the manuscript. Table S4: Represents top 20 pathway impacts based on the metabolites identified in the cell-free *L. plantarum* metabolites. Data corresponds to Figure 2C of the manuscript.
