# Supplementary material for: Chemically Defined Lactobacillus plantarum Cell-Free Metabolites Demonstrate Cytoprotection in HepG2 Cells through Nrf2-Dependent Mechanism
Source: Antioxidants (Basel). 2023 Apr 14;12(4):930. doi: 10.3390/antiox12040930 (PMC10136174; doi:10.3390/antiox12040930)
Supplement: Supplementary file 1 [file antioxidants-12-00930-s001.zip › antioxidants-2317934-supplementary.pdf]

Chemically defined *Lactobacillus plantarum* cell-free metabolites exerts cytoprotection in HepG2 cells through Nrf2-depended mechanisms

Supplementary Table S1. Chemical composition of cell-free *L. plantarum* metabolites.

S.No.	Compound Name	Area	RT	MW	M/Z	Formula	Probability (%)	Abundance (%)
1	3-Methyl-2-butenal	34504	5.2155	84.12	84 (H_4O_3S); 55 (H_7OS)	C_5H_8O	51.6	0.23
2	Glycine	38208	6.5905	75.07	75 ($C_2H_5NO_2$)	$C_2H_5NO_2$	88	0.26
3	Indole-3-acetamide	102794	6.71	174.2	130 ($C_7H_2N_2O$); 174 ($C_{10}H_{10}N_2O$)	$C_{10}H_{10}N_2O$	67.3	0.70
4	L-Alanine	183162	6.8435	89.09	74 ($C_2H_4NO_2$); 89 ($C_3H_7NO_2$)	$C_3H_7NO_2$	96	1.24
5	2-Hydroxyethylamine	25147	7	61.08	61 (C_2H_7NO)	C_2H_7NO	85.7	0.17
6	2,6-Dihydroxypyridine	38569	7.471	111.1	68 (C_3O_2); 111 ($C_5H_5NO_2$)	$C_5H_5NO_2$	67.8	0.26
7	N,N-Dimethylglycine	529103	8.329	103.12	58 (CNO_2); 103 ($C_4H_9NO_2$)	$C_4H_9NO_2$	70.4	3.58
8	Phosphonic acid	11254	10.29	81.996		H_3O_3P	49.1	0.08
9	Benzenesulfonamide	26209	10.303	157.19	77 (CHO_2S); 51 (H_3OS)	$C_6H_7NO_2S$	70.4	0.18
10	Ethyl N,N-diethylcarbamate	475809	10.5425	145.2	58 (CNO_2); 130 ($C_6H_{12}NO_2$)	$C_7H_{15}NO_2$	87.9	3.22
11	1,3-Dimethoxy-propane	7688	11.171	104.15	72 ($C_3H_4O_2$); 57 (C_2HO_2)	$C_5H_{12}O_2$	3.28	0.05
12	Acetic acid	83636	11.2625	60.05	60 ($C_2H_4O_2$)	$C_2H_4O_2$	46.9	0.57
13	L-Valine	466883	11.646	117.15	72 ($C_2H_2NO_2$); 57 (C_2HO_2)	$C_5H_{11}NO_2$	97.6	3.16
14	L-Leucine	434428	13.8725	131.17	86 ($C_3H_4NO_2$); 74 ($C_2H_4NO_2$)	$C_6H_{13}NO_2$	90.1	2.94
15	Isophthalic acid	15666	14.055	166.13	149 (C_7HO_4); 166 ($C_8H_6O_4$)	$C_8H_6O_4$	68.6	0.11
16	Thiazolidine-4-carboxylic acid	91416	14.0815	133.17	88 (C_2O_2S);61 ($CHOS$)	$C_4H_7NO_2S$	176.8	0.62
17	2-Ethylhexanoic acid	264171	14.086	144.21	73 ($C_3H_5O_2$); 88 ($C_4H_8O_2$)	$C_8H_{16}O_2$	92.4	1.79

18	l-Isoleucine	300037	14.526	131.17	86 ($C_3H_4NO_2$); 75 ($C_2H_5NO_2$); 57(C_2HO_2)	$C_6H_{13}NO_2$	88.4	2.03
19	Phosphoric acid	20832	14.601	97.9952		H_3O_4P	54.3	0.14
20	Pentanoic acid	202616	16.289	102.13	60 ($C_2H_4O_2$); 73 ($C_3H_5O_2$)	$C_5H_{10}O_2$	71.4	1.37
21	2-Propenoic acid	1076	16.374	72.06	72 ($C_3H_4O_2$); 55 (C_3H_3O)	$C_3H_4O_2$	35.8	0.01
22	Hexanoic acid	93896	16.4565	116.16	60 ($C_2H_4O_2$); 73 ($C_3H_5O_2$)	$C_6H_{12}O_2$	52.1	0.64
23	Benzoic acid	54323	16.672	122.12	105 (C_6HO_2); 122 ($C_7H_6O_2$); 77 (C_5HO)	$C_7H_6O_2$	52.9	0.37
24	Glycerol	4264296	17.5325	92.09	61 (CHO_3)	$C_3H_8O_3$	80.2	28.84
25	Decane, 3-ethyl-3-methyl-	132082	17.58	184.36	57 (C_4H_9); 85 (C_6H_{13})	$C_{13}H_{28}$	85.6	0.89
26	L-Threonine	533068	18.081	119.12	57 (C_2HO_2); 75 ($CHNO_3$)	$C_4H_9NO_3$	97.6	3.61
27	Hexane, 3,3-dimethyl-	15496	18.217	114.23	57 (C_4H_9); 71 (C_5H_{11}); 85 (C_6H_{13})	C_8H_{18}	32.9	0.10
28	Nonane, 1-iodo-	29086	18.23	254.15	71 (C_5H_{11}); 57 (C_4H_9)	$C_9H_{19}I$	95.4	0.20
29	Butanedioic acid	452489	18.6935	118.09	55 (C_3H_3O); 74 ($C_2H_2O_3$); 100 (C_3O_4)	$C_4H_6O_4$	87.5	3.06
30	Propanetriol, 2-methyl-	13226	18.7615	106.12	75 ($C_2H_3O_3$)	$C_4H_{10}O_3$	41	0.09
31	Dodecane, 4,6-dimethyl-	63250	18.9465	198.39	57 (C_4H_9); 71 (C_5H_{11})	$C_{14}H_{30}$	10.8	0.43
32	Undecane, 4,7-dimethyl-	125230	18.9545	184.36	57 (C_4H_9); 71 (C_5H_{11})	$C_{13}H_{28}$	74.5	0.85
33	Dodecanedioic acid	77545	20.4295	230.3	98 ($C_4H_2O_3$); 84 (C_3O_3)	$C_{12}H_{22}O_4$	58.7	0.52
34	l-Aspartic acid	24180	21.802	133.1	88 (C_2O_4); 70 (C_2NO_2)	$C_4H_7NO_4$	88.8	0.16
35	Aminomalonic acid	25056	22.9545	119.08	73 (C_2HO_3)	$C_3H_5NO_4$	42.3	0.17
36	2-Propenamide	7696	23.4035	71.08	71 (C_3H_5NO); 55 (C_2HNO)	C_3H_5NO	71	0.05
37	Undecane, 4-methyl	9567	23.417	170.33	57 (C_4H_9)	$C_{12}H_{26}$	91.72	0.06
38	Decane, 2,9-dimethyl-	156844	23.529	170.33	57 (C_4H_9)	$C_{12}H_{26}$	72.55	1.06
39	Butanoic acid	22023	23.724	88.11	60 ($C_2H_4O_2$)	$C_4H_8O_2$	46.4	0.15
40	3,4-Methylenedioxymandelic acid	34405	23.879	196.16	93 (CHO_5); 65 (HO_4); 151 ($C_7H_3O_4$)	$C_9H_8O_5$	49.2	0.23
41	tert-Butyl methyl carbonate	14630	23.9415	132.16	57 (C_2HO_2); 73 (C_2HO_3)	$C_6H_{12}O_3$	83.1	0.10
42	Nonane, 3,7-dimethyl-	16041	24.417	156.31	57 (C_4H_9); 71 (C_5H_{11})	$C_{11}H_{24}$	42.8	0.11
43	5-Oxoproline	1213744	24.6275	129.11	84 (C_3O_3)	$C_5H_7NO_3$	22.3	8.21

44	L-Proline	930791	25.819	115.13	70 (C_2NO_2)	$C_5H_9NO_2$	57.2	6.30
45	3-Hexene, 3,4-dimethyl-	24551	26.3065	112.21	55 (C_4H_7); 83 (C_6H_{11})	C_8H_{16}	27.2	0.17
46	2-Ethylhexyl 2-ethylhexanoate	17150	26.31	256.42	57 (C_2HO_2); 70 ($C_3H_2O_2$)	$C_{16}H_{32}O_2$	33.8	0.12
47	Mercaptosuccinic acid	242551	27.04	150.16	104 (C_2O_3S); 132 (C_3O_4S); 59 (C_2H_3S)	$C_4H_6O_4S$	87.4	1.64
48	Thiomalic acid	13859	27.079	150.16	104 (C_2O_3S); 132 (C_3O_4S); 59 (C_2H_3S)	$C_4H_6O_4S$	39.3	0.09
49	Dodecanoic acid	133510	27.6035	200.32	73 ($C_3H_5O_2$); 60 ($C_2H_4O_2$)	$C_{12}H_{24}O_2$	92.8	0.90
50	α-Aminoadipic acid	33974	28.2015	161.16	98 (CH_8NO_4); 55 (C_2HNO)	$C_6H_{11}NO_4$	32.9	0.23
51	Arabinitol	68519	29.2355	152.15	61 (CHO_3); 74 ($C_2H_2O_3$)	$C_5H_{12}O_5$	61.4	0.46
52	Dodecane	97173	29.7605	170.33	57 (C_4H_9); 71 (C_5H_{11})	$C_{12}H_{26}$	45.06	0.66
53	Octane, 2,7-dimethyl-	13926	29.973	142.28	57 (C_4H_9)	$C_{10}H_{22}$	42.7	0.09
54	Decanoic acid	37990	30.6795	172.26	60 ($C_2H_4O_2$); 73 ($C_3H_5O_2$)	$C_{10}H_{20}O_2$	45.91	0.26
55	Hexane, 3-ethyl-	24353	30.706	114.23	85 (C_6H_{13})	C_8H_{18}	58.08	0.16
56	Levoglucosan	14447	31.587	162.14	60 (CO_3); 73 (C_2HO_3)	$C_6H_{10}O_5$	30.6	0.10
57	Lauric acid	88840	31.795	220.32	73 ($C_3H_5O_2$); 60 ($C_2H_4O_2$)	$C_{12}H_{24}O_2$	76.5	0.60
58	2-Butenedioic acid	38002	32.2645	116.07	72 (C_2O_3); 54 (C_3H_2O)	$C_4H_4O_4$	76.4	0.26
59	Benzenepropanoic acid	70762	33.045	150.17	91 (C_6H_3O); 104 (C_6O_2)	$C_9H_{10}O_2$	54.7	0.48
60	Nonane, 5-methyl-5-propyl-	37363	34.291	184.36	71 (C_5H_{11}); 57 (C_4H_9); 85 (C_6H_{13})	$C_{13}H_{28}$	58.2	0.25
61	Hexadecanoic acid	841211	36.123	256.42	73 ($C_3H_5O_2$); 60 ($C_2H_4O_2$)	$C_{16}H_{32}O_2$	97	5.69
62	Octadecanoic acid	53012	39.847	284.5	73 ($C_3H_5O_2$); 129 ($C_7H_{13}O_2$)	$C_{18}H_{36}O_2$	91.8	0.36
63	Sucrose	922535	46.322	342.3	73 (C_2HO_3); 57 (C_2HO_2)	$C_{12}H_{22}O_{11}$	56.9	6.24
64	α-D-Glucopyranoside	156720	46.492	179.15		$C_6H_{11}O_6$	76.1	1.06
65	9-Acridanone	166237	51.276	195.22	195 ($C_{13}H_9NO$); 167 ($C_{11}H_5NO$)	$C_{13}H_9NO$	42.3	1.12
66	Ekatetrone acid	25032	51.3375	368.3	309 ($C_{15}HO_8$); 281 ($C_{12}H_9O_8$)	$C_{19}H_{12}O_8$	54.5	0.17

Supplementary Table S2. Chemical sub-class enrichment data of cell-free *L. plantarum* metabolites. Data corresponds to Figure 2A of the manuscript.

Metabolite Set	Total	Hits	Expect	P value	Holm P	FDR
Saturated Fatty Acids	38	8	0.00765	6.24E-23	6.53E-20	3.71E-20
Amino acids	277	11	0.0558	7.10E-23	7.41E-20	3.71E-20
Dicarboxylic acids	140	4	0.0282	2.12E-08	2.21E-05	7.39E-06
Disaccharides	9	2	0.00181	1.42E-06	0.00148	3.72E-04
Sugar alcohols	12	2	0.00242	2.61E-06	0.00272	5.45E-04
Pyrroline carboxylic acids	1	1	2.01E-04	2.01E-04	0.21	0.0263
Non-metal phosphates	1	1	2.01E-04	2.01E-04	0.21	0.0263
Saturated hydrocarbons	1	1	2.01E-04	2.01E-04	0.21	0.0263
Carboximidic acids	2	1	4.03E-04	4.03E-04	0.418	0.0468
1,2-aminoalcohols	3	1	6.04E-04	6.04E-04	0.626	0.0574
Oxepanes	3	1	6.04E-04	6.04E-04	0.626	0.0574
Benzodioxoles	4	1	8.06E-04	8.05E-04	0.834	0.0702
Straight chain Fatty Acids	6	1	0.00121	0.00121	1	0.0972
TCA acids	9	1	0.00181	0.00181	1	0.135
Benzoic acids	24	1	0.00483	0.00482	1	0.336
Branched Fatty Acids	38	1	0.00765	0.00763	1	0.499
Hydrocarbons	52	1	0.0105	0.0104	1	0.641
Carbonyl compounds	91	1	0.0183	0.0182	1	1
Unsaturated Fatty Acids	911	1	0.183	0.168	1	1
Hippuric acids	1470	1	0.296	0.257	1	1

Supplementary Table S3. Represents KEGG pathway enrichments based on the metabolites identified in the cell-free *L. plantarum* metabolites. Data corresponds to Figure 2B of the manuscript.

Metabolite Set	Total	Hits	Expect	P value	Holm P	FDR
Aminoacyl-tRNA biosynthesis	48	9	0.75	1.21E-08	1.01E-06	1.01E-06
Valine, leucine and isoleucine biosynthesis	8	4	0.125	3.09E-06	2.56E-04	1.30E-04
Alanine, aspartate and glutamate metabolism	28	3	0.438	0.0085	0.697	0.238
Glycine, serine and threonine metabolism	33	3	0.516	0.0135	1	0.282
Butanoate metabolism	15	2	0.234	0.0217	1	0.317
Valine, leucine and isoleucine degradation	40	3	0.625	0.0227	1	0.317
Starch and sucrose metabolism	18	2	0.281	0.0307	1	0.323
Pantothenate and CoA biosynthesis	19	2	0.297	0.034	1	0.323
Fatty acid biosynthesis	47	3	0.734	0.0346	1	0.323
Phenylalanine, tyrosine and tryptophan biosynthesis	4	1	0.0625	0.0611	1	0.484
Galactose metabolism	27	2	0.422	0.0648	1	0.484
Glutathione metabolism	28	2	0.438	0.0691	1	0.484
Glyoxylate and dicarboxylate metabolism	32	2	0.5	0.0873	1	0.564
Biosynthesis of unsaturated fatty acids	36	2	0.562	0.107	1	0.641
Ubiquinone and other terpenoid-quinone biosynthesis	9	1	0.141	0.132	1	0.742
Phenylalanine metabolism	10	1	0.156	0.146	1	0.767
Arginine biosynthesis	14	1	0.219	0.199	1	0.94
Nicotinate and nicotinamide metabolism	15	1	0.234	0.211	1	0.94
Histidine metabolism	16	1	0.25	0.224	1	0.94
Glycerolipid metabolism	16	1	0.25	0.224	1	0.94
Pentose and glucuronate interconversions	18	1	0.281	0.248	1	0.988
Citrate cycle (TCA cycle)	20	1	0.312	0.272	1	0.988
Selenocompound metabolism	20	1	0.312	0.272	1	0.988
beta-Alanine metabolism	21	1	0.328	0.283	1	0.988
Pyruvate metabolism	22	1	0.344	0.295	1	0.988
Propanoate metabolism	23	1	0.359	0.306	1	0.988
Lysine degradation	25	1	0.391	0.328	1	1
Glycolysis / Gluconeogenesis	26	1	0.406	0.338	1	1
Porphyrin and chlorophyll metabolism	30	1	0.469	0.379	1	1

Glycerophospholipid metabolism	36	1	0.562	0.436	1	1
Fatty acid elongation	38	1	0.594	0.454	1	1
Arginine and proline metabolism	38	1	0.594	0.454	1	1
Fatty acid degradation	39	1	0.609	0.463	1	1
Tyrosine metabolism	42	1	0.656	0.489	1	1
Primary bile acid biosynthesis	46	1	0.719	0.521	1	1

Supplementary Table S4. Represents top 20 pathway impacts based on the metabolites identified in the cell-free *L. plantarum* metabolites. Data corresponds to Figure 2C of the manuscript.

Pathway Name	p-value	-log(p)	Holm p	FDR	Impact
Aminoacyl-tRNA biosynthesis	6.49E-09	8.1878	5.58E-07	5.58E-07	0.34614
Valine, leucine and isoleucine biosynthesis	3.37E-04	3.4724	0.028644	0.01449	0.1
Alanine, aspartate and glutamate metabolism	0.0050066	2.3005	0.42056	0.12249	0.28
Valine, leucine and isoleucine degradation	0.0056971	2.2443	0.47286	0.12249	0.21429
Tyrosine metabolism	0.011052	1.9566	0.90625	0.19009	0.2
Glycine, serine and threonine metabolism	0.015791	1.8016	1	0.22634	0.24325
Glyoxylate and dicarboxylate metabolism	0.021558	1.6664	1	0.26023	0.09678
Nicotinate and nicotinamide metabolism	0.024536	1.6102	1	0.26023	0.05263
Butanoate metabolism	0.031155	1.5065	1	0.26023	0.07143
Cyanoamino acid metabolism	0.031155	1.5065	1	0.26023	0.2
Biosynthesis of unsaturated fatty acids	0.033285	1.4778	1	0.26023	0
Sulfur metabolism	0.04225	1.3742	1	0.27069	0
Novobiocin biosynthesis	0.049532	1.3051	1	0.27069	0.5
D-Alanine metabolism	0.049532	1.3051	1	0.27069	0.5
Glutathione metabolism	0.050361	1.2979	1	0.27069	0.0625
Starch and sucrose metabolism	0.050361	1.2979	1	0.27069	0.12122
Pantothenate and CoA biosynthesis	0.058994	1.2292	1	0.29844	0.08
Pyruvate metabolism	0.068109	1.1668	1	0.30828	0.04878
Methane metabolism	0.068109	1.1668	1	0.30828	0.16666
Monobactam biosynthesis	0.12697	0.89629	1	0.54598	0.2
